# Individual Noise-Tolerance Profiles and Neural Signal-to-Noise Ratio: Insights into Predicting Speech-in-Noise Performance and Noise-Reduction Outcomes

**DOI:** 10.3390/audiolres15040078

**Published:** 2025-07-02

**Authors:** Subong Kim, Susan Arzac, Natalie Dokic, Jenn Donnelly, Nicole Genser, Kristen Nortwich, Alexis Rooney

**Affiliations:** Department of Communication Sciences and Disorders, Montclair State University, Montclair, NJ 07043, USA; arzacs1@montclair.edu (S.A.); dokicn1@montclair.edu (N.D.); donnellyj9@montclair.edu (J.D.); gensern1@montclair.edu (N.G.); nortwichk1@montclair.edu (K.N.); rooneya@montclair.edu (A.R.)

**Keywords:** noise reduction, EEG, noise tolerance, neural SNR, speech perception, hearing aids

## Abstract

**Background/Objectives**: Individuals with similar hearing sensitivity exhibit varying levels of tolerance to background noise, a trait tied to unique individual characteristics that affect their responsiveness to noise reduction (NR) processing in hearing aids. The present study aimed to capture such individual characteristics by employing electrophysiological measures and subjective noise-tolerance profiles, and both were analyzed in relation to speech-in-noise performance and NR outcomes. **Methods**: From a sample of 42 participants with normal hearing, the neural signal-to-noise ratio (SNR)—a cortical index comparing the amplitude ratio between auditory evoked responses to target speech onset versus noise onset—was calculated, and individual noise-tolerance profiles were also derived using k-means cluster analysis to classify participants into distinct subgroups. **Results**: The neural SNR showed significant correlations with speech-in-noise performance and NR outcomes with varying strength. In contrast, noise-tolerance subgroups did not show meaningful group-level differences in either speech-in-noise or NR outcomes. The neural SNR and noise-tolerance profiles were found to be statistically independent. **Conclusions**: While the neural SNR reliably predicted perceptual performance in background noise and NR outcomes, our noise-tolerance profiles lacked sufficient sensitivity. Still, subjective ratings of individual noise tolerance are clinically accessible, and thus, integrating both physiology and subjective measures in the same cohort is a valuable strategy.

## 1. Introduction

Two individuals with similar hearing sensitivity (or audiograms) listen to the same speech sounds in a noisy place, but one follows along relatively easily while the other finds it distracting. Just as in this case, individuals inherently have different noise sensitivity or tolerance, which may affect their quality of life in general [1,2] and is further exacerbated in people who are diagnosed with hearing loss [3,4,5]. Even when a patient is properly amplified, this does not always result in perceived benefits in background noise, and many hearing aid users report a low tolerance to high or moderate levels of noise, leading to less use of hearing aids [6,7]. To address this situation, modern digital hearing aids implement various noise reduction (NR) algorithms to suppress background noise [8,9,10], but this also adds another level of complexity, as it may compromise speech quality [11,12]. Consequently, some people prefer NR and appreciate the amount of noise attenuated, whereas others dislike it due to unnatural speech [13,14,15]. These findings highlight the need to develop measures to accurately capture individual characteristics regarding their reaction to noise [6,7] and provide personalized treatment in hearing clinics [16,17,18,19]. This study defines the term *noise tolerance* to denote such individual characteristics, which may stem from the fact that some people dislike noise itself, others are sensitive to speech interference due to noise, and some find it frustrating due to the cognitive effort exerted to process speech in noise [20,21,22].

The literature shows that numerous studies have attempted to capture factors driving individual differences in noise tolerance using a variety of audiological and behavioral measures; however, these efforts have fallen short of encapsulating their full scope [13,18,23,24]. Some studies have incorporated electrophysiological measures to enhance the accuracy of capturing individual characteristics in regard to an individual’s ability to perceive speech in noise, with cortical auditory evoked potentials proving to be a useful neural predictor of speech perception in noise across various hearing sensitivity and age levels [25,26,27,28,29]. Recent progress in neural tracking of speech has shown promising advancements as an objective measure of speech intelligibility in individuals with normal and impaired hearing based on their efficiency in neural entrainment to the speech envelope [30,31,32] and as a tool for assessing the auditory pathway integrity [33] and evaluating neural benefits of NR algorithms improving speech perception and selective attention [34,35,36]. However, in addition to evaluating how individuals encode spectrotemporal features of speech sounds, existing electrophysiology studies have often overlooked their level of efficiency in suppressing background noise.

The neural signal-to-noise ratio (SNR), as defined by Kim and colleagues, represents the amplitude ratio between cortical auditory responses to noise onset and target speech onset, aiming to reflect both noise suppression efficiency and speech encoding precision within a single neural index [37,38]. The neural SNR quantifies how well an individual’s brain differentiates between target speech and background noise and aligns with the concept of a neural correlate of the target-to-masker ratio [39,40]. It serves as a potential biomarker for successful speech-in-noise comprehension, showing significant correlations with behavioral performance in the presence of noise in different hearing populations, including individuals with normal hearing [38] and cochlear implant patients [41,42]. Further, the neural SNR was significantly correlated with NR outcomes [37], with stronger correlations emerging as NR strength increased [43].

Although electrophysiological indexes such as neural SNR could be sensitive tools to predict individual speech-in-noise and NR outcomes, they are not integrated into the typical workflow in clinical settings. Our recent study combined cortical and subjective measures in the same participants to determine how well the cortical measure of noise tolerance (i.e., neural SNR) aligns with self-reported noise tolerance [43]. While both the neural SNR and self-reported noise tolerance rating were significant predictive of hearing outcomes with varying NR strength, the neural SNR showed a stronger association. We also found that the cortical and subjective measures were not significantly correlated, implying they may assess non-overlapping correlates of individual noise tolerance. The limited sensitivity of our subjective measure, the Weinstein Noise Sensitivity Questionnaire [44,45], may be attributable to its focus on a single domain of noise tolerance (i.e., noise annoyance). Mackersie, Kim [20] suggested that individual noise tolerance encompasses multiple domains, and their findings showed that noise tolerance thresholds varied across participant clusters based on such noise-tolerance domains (e.g., noise annoyance, distraction, speech interference). However, those noise-tolerance domains were not discussed in relation to speech-in-noise and NR outcomes, nor was it explored whether they relate to electrophysiological indexes such as neural SNR.

The current study aimed to examine whether subjective noise-tolerance profiles—by identifying participant subgroups based on noise-tolerance domain ratings with k-means cluster analysis—and neural SNR are reliable predictors of speech-in-noise performance and NR outcomes. This noise-tolerance profiling approach, while inherently subjective, was used to investigate whether systematically derived subgroups could provide a sensitive measure of individual noise tolerance in relation to speech-in-noise and NR outcomes. We hypothesized that neural SNR would be correlated with speech-in-noise performance and NR outcomes and that noise-tolerance profiles would reveal significant differences in those outcomes between the resulting subgroups.

## 2. Materials and Methods

### 2.1. Participants

A total of 42 adults (8 male, 34 female; mean age = 22.74 years, SD = 3.86) whose primary language of communication is American English participated in this study. All participants showed air-conduction thresholds of less than 20 dB HL at 0.5, 1, 2, and 4 kHz in both ears, with hearing symmetry within 20 dB, measured using a GSI AudioStar Pro (Grason-Stadler Inc., Littleton, MA, USA) coupled with TDH-39P supra-aural headphones (Telephonics Corporation, Farmingdale, NY, USA).

### 2.2. Task Design and Procedures

Participants were seated in a chair 0.5 m from the front monitor at eye level in a single-walled sound-attenuated booth. Auditory stimuli were delivered via ER-2 insert earphones (Etymotic Research, Elk Grove, IL, USA) monaurally to a participant’s better ear, determined by hearing thresholds averaged across 0.5, 1, 2, and 4 kHz. All experimental procedures were implemented in MATLAB (R2022b, MathWorks, Natick, MA, USA) using the Psychtoolbox-3 [46].

**Figure 1:**
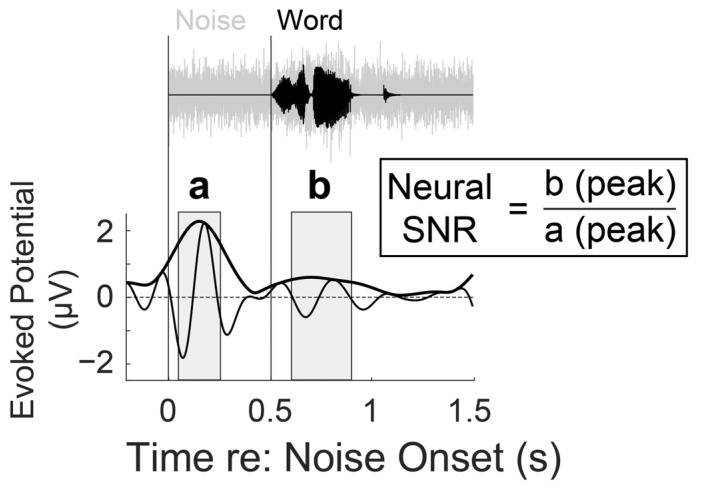
Trial structure of the speech-in-noise task. The top panel illustrates that speech-shaped noise occurs half a second prior to the presentation of a monosyllabic word (in this example, “sat”) and continues for another second. The bottom panel depicts electroencephalographic recordings averaged across the front-central channels during the task. The neural signal-to-noise ratio (SNR) for a given individual is calculated using the ratio (b/a) of the peak amplitude of temporal envelopes within a time window (gray-colored period) of 50 to 250 and 100 to 400 ms following the noise and word onset, respectively.

The first task was a speech-in-noise test in which consonant-vowel-consonant monosyllabic English words [47] and speech-shaped noise were presented at 0 dB SNR. The composite stimuli were root mean square (RMS) normalized and presented at 80 dB A. The speech-in-noise task included three experimental conditions (NR off and two NR algorithms described further in the next section). Each condition was randomly assigned one of three sets of target words, each consisting of 55 unique words, resulting in 165 non-repeating words. The first phonemes were balanced across the word sets, in terms of features of place of articulation, voicing, and manner of articulation, to minimize bias in participant responses. Each trial started with the indication of the trial number and a half-second of silence, followed by another half-second with the “+” sign on the center of the monitor. Then, speech-shaped noise was presented for 1.5 s, and a target word started 0.5 s after the noise onset (Figure 1 top panel). When the auditory stimuli presentation ended, participants pressed the number pad to answer four multiple-choice questions provided on the screen; for the target word “sat,” the other three words were “pat,” “fat,” and “that.” Participants did not receive feedback at the end of the trial. Electroencephalogram (EEG) was simultaneously recorded during the speech-in-noise task, and the details of data acquisition and preprocessing are described in a later section.

The second task was a noise-tolerance domain test examining the relative importance of three subjective domains, *noise annoyance*, *speech interference*, and *listening effort*, in noise-tolerance judgments among individuals. Individuals were verbally instructed on the definition of each domain (Table 1) at the beginning of the task and displayed on the monitor screen throughout the task. A list of ten IEEE sentences [3] was presented in speech-shaped noise at −6 dB SNR, which aimed for a moderate difficulty level at around 50% accuracy calculated based on correctly identified keywords from our preliminary data. In each trial, a sentence was presented six times, including a series of three absolute ratings and three paired comparisons for a given sentence, similar to procedures reported by Mackersie et al. (2021) [20]. In the first three repetitions for absolute ratings, participants were asked to rate each noise-tolerance domain on a scale from 1 (no annoyance/interference/effort at all) to 10 (extreme annoyance/interference/effort). In the subsequent three repetitions for paired comparisons in which each noise-tolerance domain was paired with another, participants were instructed to select the domain more responsible for intolerance of the noise, such as, “Which of the following bothered you more: noise annoyance or speech interference?” Individual (0 to 10) weighted ratings were calculated by multiplying the average absolute rating (1 to 10) and paired-comparison score (%) for each domain.

### 2.3. Quantification of NR Effects on Stimuli: SNR Enhancement and Speech Distortion

The present study implemented two Ephraim–Malah NR algorithms: one with the minimum mean square error (MMSE) estimator (henceforth called NR 1 or mild NR) [48] and the other with the Log-MMSE estimator (henceforth called NR 2 or strong NR), which employs more intense noise attenuation [49]. These modified spectral subtraction-based NR algorithms were used in digital hearing aids [50,51] and continue to underpin modern NR algorithms integrated with techniques like deep learning or deep neural networks [52,53]. Both NR algorithms divide the audio stimulus into overlapping 20 ms time frames based on a Hamming window, apply a fast Fourier transform (FFT) and calculate the magnitude spectrum of each frame, implement the spectral gain based on the estimation of a posteriori SNR in the current frame and a priori SNR in the previous frame, and apply the inverse FFT to the modified spectrum to reconstruct the signal in the time domain (for further details, refer to Cappé [54], Marzinzik [55]). The main difference between NR 1 and NR 2 algorithms lies in calculating the spectral gain; more specifically, NR 1 aims to minimize the mean square error of the *linear* spectral amplitude [48], whereas NR 2 seeks to minimize the error of the *logarithmic* spectral amplitude [49].

The accurate measurement of the two conflicting NR effects (SNR enhancement and speech distortion) on auditory stimulus is a prerequisite for investigating how individual noise tolerance characteristics relate to the outcomes of NR processing [56]. First, we evaluated how much SNR enhancement was given with each NR algorithm using the phase-inversion technique [57] commonly implemented in hearing-aid studies [23,58,59]. This technique processes two noisy signals that are identical except for a phase in noise through NR algorithms. Adding or subtracting NR-processed stimuli results in post-NR speech signal and noise, respectively, to be used to calculate the SNR changes compared to pre-NR SNR. The current study revealed that NR 1 provides 3.5 dB, and NR 2 provides 5 dB enhancement in SNR based on non-weighted RMS measurements. Second, we also evaluated the extent to which speech distortion occurred due to NR algorithms using the magnitude-squared coherence method [60], commonly used in signal-processing studies [61,62,63]. The current study estimated the spectral coherence between pre- and post-NR *speech* stimuli to make a coherence value between 0 (totally different) and 1 (identical), showing 0.60 for NR 1 and 0.26 for NR 2, averaging up to 22 kHz.

Overall, our assessment of NR effects on auditory stimulus revealed that NR 1 provides relatively less SNR benefit but introduces lower spectral distortion, whereas NR 2 offers more SNR enhancement but at the cost of more severe distortion.

### 2.4. Data Acquisition and Preprocessing

EEG data were collected at a sampling rate of 4096 Hz during the speech-in-noise task following the 10–20 international layout using a 64-channel BioSemi ActiveTwo system (BioSemi B.V., Amsterdam, The Netherlands). Electrode data were preprocessed offline using MATLAB (R2022b, MathWorks, Natick, MA, USA). The data were bandpass-filtered between 1 and 50 Hz, implementing a zero-phase finite impulse response (FIR) filter with the two-pass approach (forward and reverse filtering) to prevent phase distortion [64], and were re-referenced to the average of both mastoids. Continuous EEG was then epoched from −0.5 to 2.5 s, referring to the onset of noise in each trial. Each epoch was baseline-corrected by subtracting the mean amplitude during the 200 ms pre-stimulus period and down-sampled to 256 Hz. The Infomax ICA algorithm in EEGLAB [65,66] was used to separate epoched data into statistically independent components. Based on the pattern of spatial topography, time courses, and power spectrum, ocular artifacts (e.g., blinks and saccades) were identified and removed by visual inspection. Cleaned epochs corresponding to each experimental condition were averaged to compute event-related potentials (ERPs) per condition in each electrode channel.

The neural SNR was defined as the ratio of the ERP amplitude to target speech onset versus amplitude to noise onset, where higher neural SNR indicates more efficient cortical differentiation between speech and noise. To calculate the neural SNR, the temporal envelopes of mean ERPs over the front-central channels (FZ, FCz, FC1, FC2, and Cz) were analyzed by implementing a zero-phase FIR bandpass filter between 2 and 7 Hz, applying the Hilbert transform, and computing the magnitude over time to capture the full dynamics of neural activity instead of solely focusing on a single component. The current study used ERP temporal envelopes from the “*NR-off*” condition to compute the neural SNR in the dB scale by comparing the maximum amplitude of the temporal envelope between 100 and 400 ms after the word onset to one between 50 and 250 ms after the noise onset (Figure 1 bottom panel).

### 2.5. Statistical Analysis

The current study performed a one-way repeated-measures ANOVA to analyze behavioral accuracy performance. Then, correlations of neural SNR with speech-in-noise performance and NR-driven accuracy changes (Δaccuracy) were evaluated using Pearson analysis. Further, based on our second task (noise-tolerance domain test) results, we implemented k-means cluster analysis to identify noise-tolerance subgroups among participants using the weighted ratings of noise annoyance, speech interference, and listening effort. Based on Mackersie, Kim [20] and the elbow method, the initial optimal number of clusters was determined as three. In each noise-tolerance domain, a one-way ANOVA and post hoc analysis were conducted to check whether all comparisons were significant and, if not, to reevaluate the number of clusters (i.e., two). The silhouette score comparison and additional two-sample *t*-test were used to validate the adjusted two-cluster solution. After the clustering adjustment was finalized, a two-sample *t*-test was conducted to compare neural SNR, speech-in-noise performance, and NR 1- and NR 2-driven accuracy changes between the two clusters of participants.

## 3. Results

### 3.1. Behavioral Performance

Behavioral accuracy performance largely varied among participants (NR off: mean = 77.75%, median = 79.09%, SD = 7.39%; NR 1: mean = 80.30%, median = 81.82%, SD = 8.04%; NR 2: mean = 73.51%, median = 74.55%, SD = 7.96%). A one-way repeated-measures ANOVA revealed that NR significantly affected behavioral accuracy (*F*_2,82_ = 13.00, *p* < 0.001). Post hoc paired-samples *t*-tests with Bonferroni corrections showed that although NR 1 was not significantly different from NR off (*t*(41) = −1.93, adjusted *p* = 0.18), a significant difference was observed between NR off and NR 2 (*t*(41) = 3.14, adjusted *p* = 0.0094), and thus, NR 1 and NR 2 also significantly differed (*t*(41) = 4.98, adjusted *p* < 0.001). These findings that only a fraction of people improved their behavioral performance in noise, with 22 people (52%) from NR 1 and 10 people (24%) from NR 2, are consistent with the literature reporting no improvement in performance with NR [9,67] or a decline in performance [11,68] despite documented benefits of NR in reducing listening effort and improving the listening experience [9,24,50,67,69,70,71].

Nevertheless, it should be noted that among those who showed enhancement in performance with NR 1, 9 out of 22 individuals (41%) also benefited from NR 2. Similarly, among those who showed a decline in performance with NR 1, 13 out of 16 (81%) exhibited the same trend with NR 2. The significant correlation (*r* = 0.48, *p* = 0.0013) between NR-driven accuracy changes (i.e., NR 1 minus NR off vs. NR 2 minus NR off) further supports inherent *individual characteristics* that may play a role in determining their NR outcomes. Therefore, the following sections show the relationship between individual noise tolerance measures and three outcome measures (NR-off accuracy and NR 1- and NR 2-driven accuracy changes).

### 3.2. Relationship Between Neural SNR and Speech-in-Noise Performance and NR Outcomes

**Figure 2:**
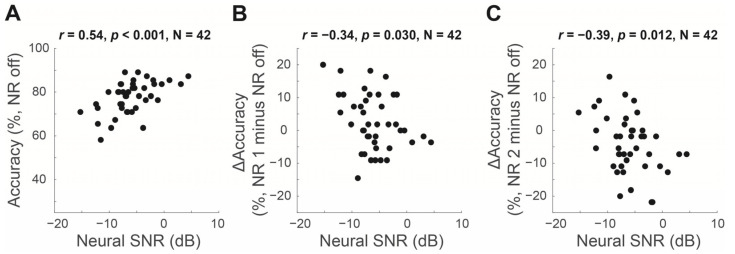
Correlation of neural signal-to-noise ratio (SNR) with noise reduction (NR)-off accuracy (i.e., speech-in-noise performance) and NR 1- and NR 2-driven accuracy changes (Δaccuracy). Higher levels of neural SNR are associated with higher levels of NR-off accuracy (**A**), whereas lower levels of neural SNR correspond to higher levels of NR 1-driven ((**B**) NR 1 minus NR off) and NR 2-driven accuracy changes ((**C**) NR 2 minus NR off).

Pearson correlation analysis examined the relationships between the neural SNR and NR-off accuracy (i.e., speech-in-noise performance) and NR 1- and NR 2-driven accuracy changes (Δaccuracy), respectively. The neural SNR showed significant positive correlations with NR-off accuracy (*r* = 0.54, *p* < 0.001) and significant negative correlation with behavioral accuracy changes by NR 1 (*r* = −0.34, *p* = 0.030) and NR 2 (*r* = −0.39, *p* = 0.012) (Figure 2), suggesting a direct relationship between neural SNR and NR-off accuracy such that higher neural SNR corresponds to higher performance in noise, and inverse relationships between neural SNR and NR-driven accuracy changes, where an individual’s lower neural SNR is potentially associated with higher perceptual benefits from NR 1 and NR 2.

### 3.3. Noise-Tolerance Profiles and Speech-in-Noise Performance and NR Outcomes

**Figure 3:**
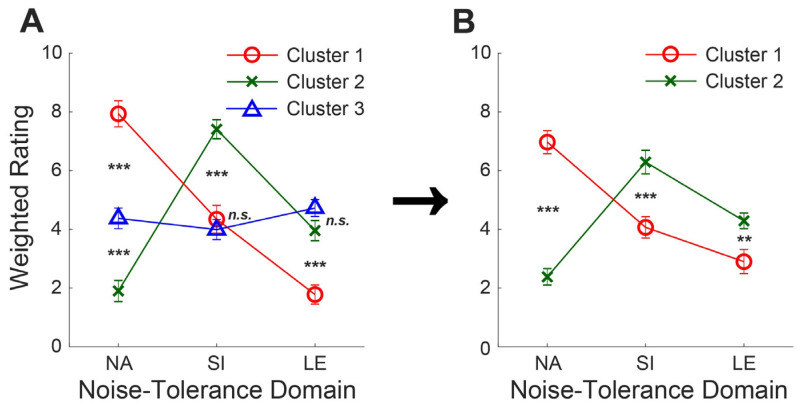
Mean weighted rating, ±1 SEM, for each noise-tolerance domain: noise annoyance (NA), speech interference (SI), and listening effort (LE). (**A**) In the initial k-means cluster analysis with three clusters of participants, not all pairwise comparisons are significantly different, prompting a reassessment of the number of clusters. (**B**) The adjustment to two clusters of participants results in significantly different ratings between the clusters for each domain. ***, Significant at *p* < 0.001; **, significant at *p* < 0.01; *n.s.*, not significant.

K-means cluster analysis initially assigned each individual to one of *three* clusters. Three separate ANOVAs showed a significant group effect for each domain (noise annoyance: *F*_2,39_ = 58.00, *p* < 0.001, speech interference: *F*_2,39_ = 25.43, *p* < 0.001, listening effort: *F*_2,39_ = 21.81, *p* < 0.001). Post hoc analysis (Tukey’s test) revealed that a couple of pairwise comparisons were not significant (speech interference between cluster 1 and 3: mean difference = −0.35, adjusted *p* = 0.77, 95% CI [−1.66, 0.96], listening effort between cluster 2 and 3: mean difference = 0.77, adjusted *p* = 0.20, 95% CI [−0.29, 1.83]) (Figure 3A), so the current study reduced the number of clusters to *two* to generate more distinct noise-tolerance subgroups. Then, the silhouette score was recalculated to ensure that the new two-cluster solution was valid; the silhouette score of 0.35 for three clusters and 0.37 for two clusters were found. An additional two-sample *t*-test revealed that the two resulting clusters were significantly distinct for each domain (noise annoyance: *t*(34.90) = 9.51, *p* < 0.001, speech interference: *t*(39.89) = −4.09, *p* < 0.001, listening effort: *t*(33.06) = −2.84, *p* = 0.0077) (Figure 3B).

In conclusion, 20 individuals were assigned to cluster 1, whose noise-tolerance judgments were heavily weighted on noise annoyance, and 22 individuals were assigned to cluster 2, whose judgments were more driven by speech interference or listening effort.

**Figure 4:**
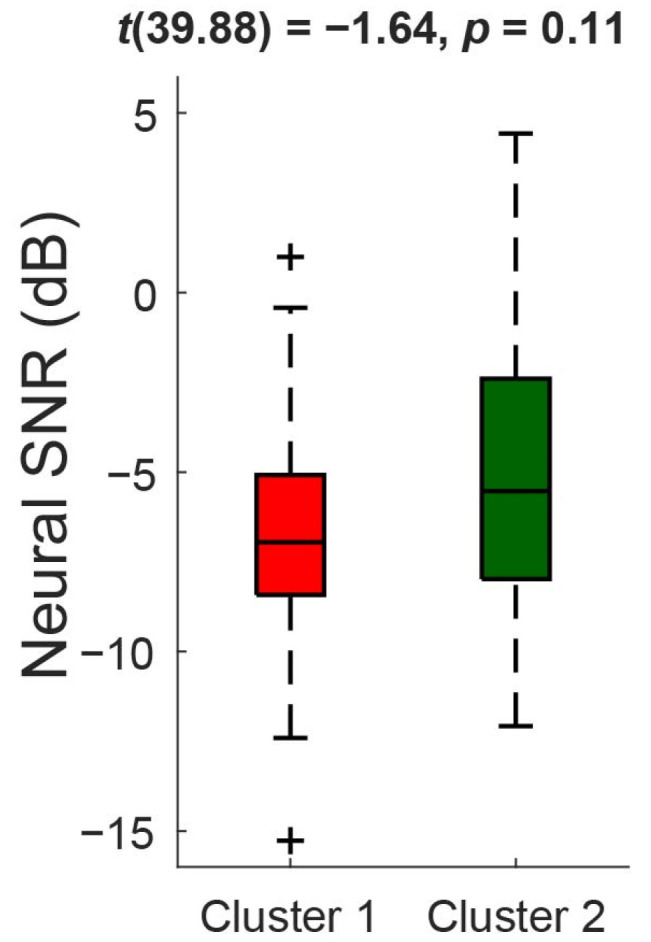
Boxplots of neural signal-to-noise ratio (SNR) compared between two clusters of participants. The center of the box plot marks the median, and the edges indicate the 25th and 75th percentile. A two-sample *t*-test was performed.

**Figure 5:**
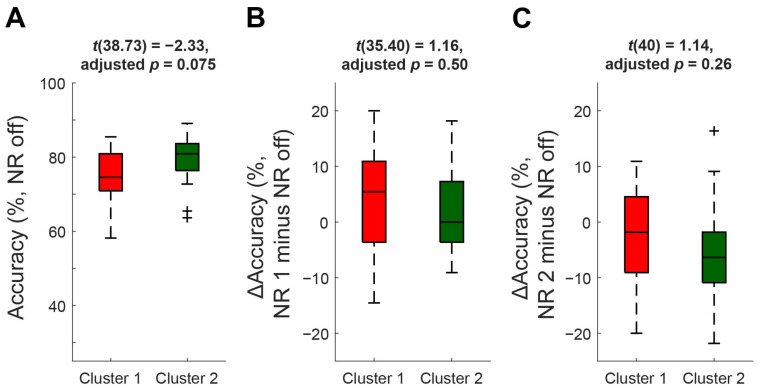
Boxplots of behavioral accuracy in the presence of noise ((**A**) noise reduction (NR) off) and accuracy changes (Δaccuracy) driven by NR 1 ((**B**) NR 1 minus NR off) and NR 2 ((**C**) NR 2 minus NR off) compared between two clusters of participants. The center of the box plot indicates the median, and the edges represent the 25th and 75th percentile. Three separate two-sample *t*-tests with Holm–Bonferroni corrections were performed.

No significant difference was found in neural SNR between the two clusters of participants (*t*(39.88) = −1.64, *p* = 0.11) (Figure 4), revealing a lack of a relationship between cortical and subjective measures used in the present study. Further, there were no significant differences in NR-off accuracy (*t*(38.73) = −2.33, adjusted *p* = 0.0075) and accuracy changes by NR 1 (*t*(35.40) = 1.16, adjusted *p* = 0.50) and NR 2 (*t*(40) = 1.14, adjusted *p* = 0.26) between the two clusters after applying the Holm–Bonferroni correction (Figure 5), indicating that the cluster solution applied in the current study may have limited sensitivity to group differences in these outcome measures.

## 4. Discussions

Our findings in behavioral performance were in line with the literature, reinforcing the idea that NR may not enhance speech intelligibility in noise [9,11,67,68]. However, our data revealed considerable individual variability, with notable consistency in performance changes across different NR strengths, indicating the need for more sensitive measures to capture individual noise tolerance characteristics responsible for their reaction to noise and NR processing. The present electrophysiological results are in line with our prior studies, further substantiating the role of the neural SNR in predicting speech-in-noise performance and NR outcomes across different experimental conditions and hearing populations [37,38,41,42,43].

Noise-tolerance profiles based on k-means cluster analysis did not appear sufficiently sensitive to capture individual noise tolerance in relation to speech-in-noise performance and NR outcomes. Although group differences were not statistically significant, cluster 1 (noise annoyance group) exhibited lower behavioral accuracy in noise and greater benefit from NR than cluster 2. This finding aligns with our prior electrophysiological models [37,38,43], suggesting that individuals with poor neural SNR show lower speech-in-noise performance and benefit more from external NR processing. In fact, cluster 1 had lower neural SNR than cluster 2 despite no statistical significance. This trend aligns with our previous findings [37,38], indicating that people with lower neural SNR showed poor neural suppression of irrelevant auditory input. Similarly, in their noise-tolerance profile study, Mackersie et al. found that the noise-annoyance group was the least tolerant of noise and had the highest noise-tolerance threshold [20]. Participants in cluster 1 in the present study appeared to be more reactive to noise and, consequently, may have less effective suppression of background noise, resulting in lower neural SNR. In contrast, we interpret relatively higher neural SNR in cluster 2, whose judgments were mainly determined by speech interference and listening effort, as reflecting more successful auditory gain control [39,40] that may enhance cortical differentiation between speech and noise in this group of participants. This is consistent with prior findings linking higher neural SNR to higher speech-in-noise performance and fewer NR benefits [37,38,43].

Given that the neural SNR encompasses noise-onset evoked responses and responses to target speech onset [37,38,43], and our noise-tolerance domains also involve noise annoyance and speech interference [20], we expected some degree of correspondence between neural SNR and subjective noise-tolerance profiles. However, no significant relationship was observed between them. One possible explanation for the lack of association is that, although both measures include components related to how an individual reacts to *noise* and *speech*, the nature of these components may substantially differ across physiological and subjective measures. For instance, findings from Kim, Schwalje [38] indicate that variability in neural SNR may stem from selective attentional efficacy reflected in different noise-onset evoked response patterns between good and poor performers on speech-in-noise tasks, which is likely not reflected in the noise-tolerance profiles in the present study. Alternatively, our noise-tolerance domain test may not have been as sensitive as that of Mackersie, Kim [20]. For example, the domain of listening effort was not clearly distinguishable from the speech interference domain, possibly due to the less challenging noise level involved in the present study. It is also possible that the relatively homogeneous nature of our sample (i.e., individuals with normal hearing) may have limited the variability necessary for distinct participant subgroup differentiation.

An exploratory cluster analysis was conducted using the same k-means clustering method as the original analysis, this time solely based on speech interference and listening effort criteria (Supplementary Figure S1A). Interestingly, we found that the speech-interference group showed greater benefit from NR 1 (*t*(39.01) = 3.19, adjusted *p* = 0.0084) compared to the listening-effort group (Supplementary Figure S2B). However, the speech-interference group exhibited higher neural SNR than the listening-effort group (Supplementary Figure S1B), which was not consistent with the neural SNR pattern observed in our original analysis and previous findings [37,38,43], where higher neural SNR was associated with lower NR benefits. This implies that the speech-interference group may have adequate early sensory encoding (as reflected in high neural SNR) but still struggle in the presence of noise and appreciate NR due to limitations from their higher-level linguistic or cognitive functions, such as working memory and speech parsing [72,73,74]. Taken together with our original analysis, these results suggest that individual noise tolerance may stem from one reflecting sensory suppression and auditory encoding efficiency (as captured by the noise annoyance-based clustering and its association with neural SNR) and another reflecting speech-specific cognitive processing demands and perceived effort (as captured by the exploratory cluster analysis with speech interference and listening effort). To test this framework further, future studies should consider real-time assessments of cortical and subjective noise tolerance. This would allow for an online assessment of noise tolerance collected concurrently with EEG recordings, providing insights into cortical dynamics of how individuals with different noise-tolerance profiles process speech sounds embedded in noise. This multimodal approach may facilitate the identification of more clearly defined subgroups and reveal corresponding differences in neural signatures across those groups.

Despite the limitations, the present study provides a valuable step toward bridging subjective and physiological measures of individual noise tolerance and understanding their relationship to speech-in-noise and NR outcomes. This approach aligns well with previous studies emphasizing the value of integrating behavioral and physiological measures [27,28,29,75,76]. Our findings support the utility of the neural SNR in assessing individual differences in noise tolerance, yet self-reported ratings from patients remain clinically relevant and widely available, highlighting their ongoing importance in practice. While this remains a long-term goal, future work should focus on advancing subjective measures with better sensitivity and developing integrative frameworks involving both physiological and subjective measures to enhance diagnostic and intervention outcomes.

## Figures and Tables

**Table 1:** Definitions of the three noise-tolerance domains.

Domain	Definition
Noise annoyance	The way the noise sounds is annoying.
Speech interference	The noise causes me to miss portions of what I need to hear.
Listening effort	The noise makes me put in more effort to hear.

## Data Availability

The data that support the findings of the present study have been made publicly available in the Mendeley Data at https://dx.doi.org/10.17632/8j3y6t32y2.1 and accessed on 28 November 2024.

## Supplementary Materials

The following supporting information can be downloaded at: https://www.mdpi.com/article/10.3390/audiolres15040078/s1, Figure S1: Exploratory cluster grouping based on speech interference (SI) and listening effort (LE) and the neural signal-to-noise ratio (SNR) compared between two clusters of participants; Figure S2: Exploratory cluster comparison of speech-in-noise performance and noise-reduction (NR) outcomes: speech interference (cluster 1) vs. listening effort (cluster 2).
