# Supplementary material for: Individual Noise-Tolerance Profiles and Neural Signal-to-Noise Ratio: Insights into Predicting Speech-in-Noise Performance and Noise-Reduction Outcomes
Source: Audiol Res. 2025 Jul 2;15(4):78. doi: 10.3390/audiolres15040078 (PMC12286289; doi:10.3390/audiolres15040078)
Supplement: Supplementary file 1 [file audiolres-15-00078-s001.zip › audiolres-3624713-supplementary.pdf]

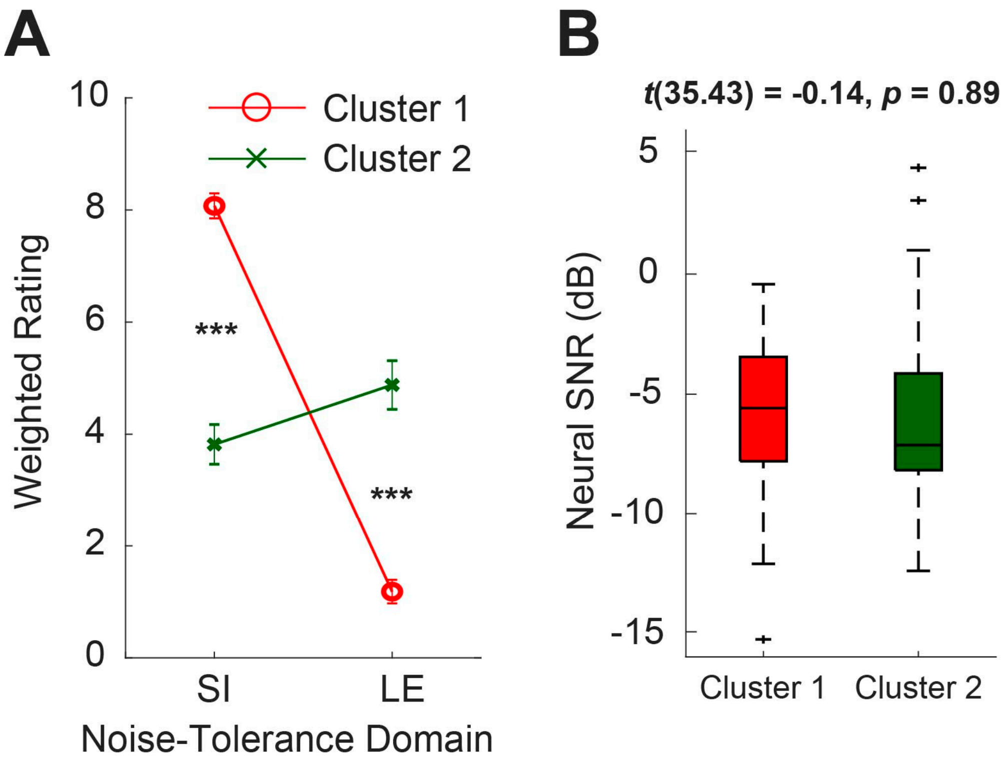

Figure S1. Exploratory cluster grouping based on speech interference (SI) and listening effort (LE) and the neural signal-to-noise ratio (SNR) compared between two clusters of participants.

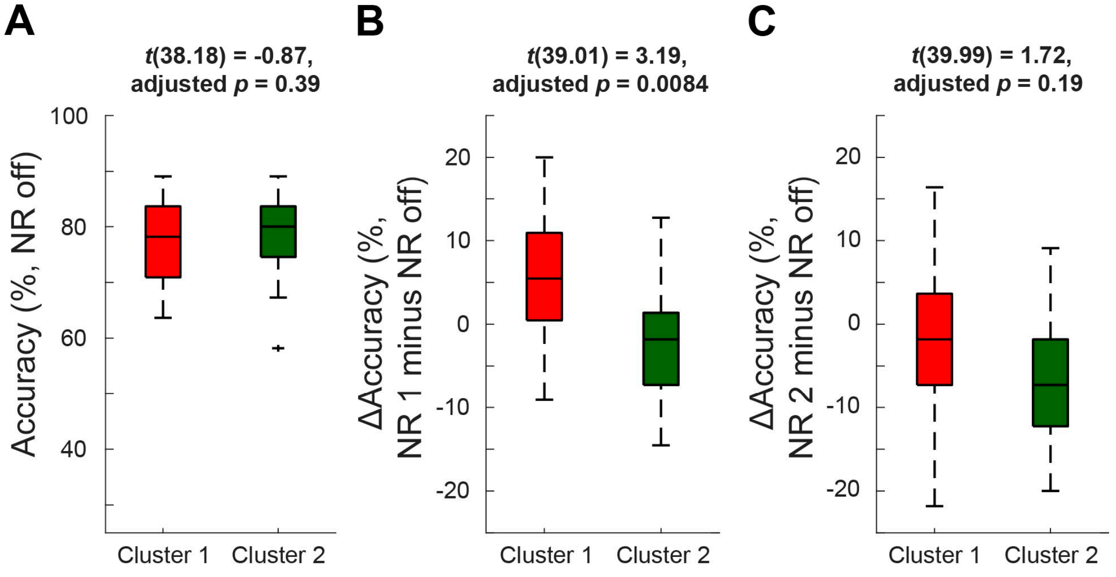

Figure S2. Exploratory cluster comparison of speech-in-noise performance and noise-reduction (NR) outcomes: speech interference (cluster 1) vs. listening effort (cluster 2).
